# Regulation of Akt(ser473) phosphorylation by Choline kinase in breast carcinoma cells

**DOI:** 10.1186/1476-4598-8-131

**Published:** 2009-12-31

**Authors:** Boon Tin Chua, David Gallego-Ortega, Ana Ramirez de Molina, Axel Ullrich, Juan Carlos Lacal, Julian Downward

**Affiliations:** 1 Singapore OncoGenome Project, Institute of Medical Biology, A*STAR, 8A Biomedical Grove, #06-06 Immunos, 138648, Singapore; 2 Translational Oncology Unit CSIC-UAM-La Paz, Centro Nacional de Biotecnología (CNB), Darwin 3, 28049 Madrid, Spain; 3 TCD Pharma, Parque Científico de Madrid, Pabellón C, planta baja, Einstein 13, Campus de Cantoblanco, 28049 Madrid, Spain; 4 Max-Planck Institute of Biochemistry, Am Klopferspitz 18, D-82152 Martinsried, Germany; 5 Signal Transduction, Cancer Research UK London Research Institute, 44 Lincoln's Inn Fields, London WC2A 3PX, UK

## Abstract

**Background:**

The serine/threonine kinase PKB/Akt plays essential role in various cellular processes including cell growth and proliferation, metabolism and cell survival. The importance of the Akt pathway is highlighted by the mutation of various components of the pathway such as the PTEN and PI3-kinase (P110α) in human cancers. In this paper, we employed an RNA interference library targeting all human kinases to screen for kinases involved in the regulation of Akt activation, in particular serine 473 phosphorylation. Here, we transfected the MDA-MB 468 breast cell line with the human kinome siRNA library and measured Akt activation using an antibody specific for phosphoserine 473 of Akt.

**Results:**

The screen revealed that phosphorylation of Akt(ser473) can be regulated by more than 90 kinases. Interestingly, phosphorylation of Akt(ser473), but not thr308, can be severely reduced by inhibition of Choline kinase activity *via *siRNA or small molecule inhibitors. We show here that the regulation of Akt phosphorylation by Choline kinase is PI3K-independent. In addition, xenograft tumors treated with Choline kinase inhibitors demonstrated a statistically significant decrease in Akt(ser473) phosphorylation. Importantly, the reduction in phosphorylation correlates with regression of these xenograft tumors in the mouse model.

**Conclusion:**

High Choline kinase expression and activity has previously been implicated in tumor development and metastasis. The mechanism by which Choline kinase is involved in tumor formation is still not fully resolved. From our data, we proposed that Choline kinase plays a key role in regulating Akt(ser473) phosphorylation, thereby promoting cell survival and proliferation.

## Background

Akt or Protein kinase B, is a serine/threonine kinase that plays an important role in regulating a number of cellular processes such as growth, metabolism and survival (reviewed in [1]). The importance of the Akt pathway is highlighted by the mutation of various components of the pathway in human cancers such as the PTEN and PI3-kinase (P110α), which occur in more than 30% of human tumors (reviewed in [2]). In recent years, much has been invested in the search for other Akt substrates in the hope of understanding the different cellular processes controlled by Akt. Currently over fifty Akt substrates have been identified.

For Akt to achieve full activation, phosphorylation is needed at both serine 473 (ser473) of the hydrophobic tail and threonine 308 (thr308) of the activation motif, upon growth factor ligation to the receptor tyrosine kinases [3]. The extra-cellular growth signal is transduced *via *the Ras protein resulting in the activation of PI3K. The lipid kinase phosphorylates phosphatidylinositol 4,5-bisphosphate (PIP_2_) to phosphatidylinositol (3,4,5)-trisphosphate (PIP_3_) which acts as a secondary messenger to recruit Akt *via *its PH domain to the peripheral membrane. Similarly, PDK1 is also recruited *via *its PH domain to phosphorylate thr308 of Akt. To date, there are several candidate kinases fulfilling the role of PDK2, for the ser473 residue, the most likely candidate being the mTORC2 [4]. Others include DNA-PK, ILK and some PKCs [5-9].

Choline kinase (ChoK), is a lipid kinase that phosphorylates choline to generate phosphoryl choline (PCho). PCho serves as the first step in the Kennedy pathway for the generation of phosphatidylcholine [10], a major lipid component of the cellular membrane. In the last few years, high PCho and ChoK activity has been found in several human tumor types including breast, lung, colon and prostate [11,12]. There is a strong clinical correlation between ChoK expression level and tumor malignancy in breast, lung and bladder cancer [13,14]. Several reports have also demonstrated that with the inhibition of ChoK either by siRNA or small molecule inhibitors, there is a marked reduction in proliferation and mitogenic properties and a decrease in breast cancer cell viability has being reported in combination with 5-fluorouracil [15,16]. A full understanding of how this lipid kinase and its downstream substrates contribute to tumorigensis has yet to be disclosed, although some previous studies clearly correlate ChoK regulation with Rho A signaling, and transcriptome analysis of ChoK overexpression demonstrates its effects on cell cycle regulation and apoptosis impairment [17-19]. Previously, it has been shown that PCho confers mitogenic properties to mouse fibroblasts upon stimulation by PDGF or FGF [20,21].

In this work, we searched for kinases that could regulate Akt activity specifically at ser473. Using a human kinome siRNA library, we silenced individual kinases systematically in MDA-MB 468 cells to screen for candidate kinases that regulate Akt phosphorylation at this site using an indirect immunofluorescent method. In our system, MDA-MB 468 breast carcinoma cells were used for its high endogenous Akt phosphorylation in the absence of growth factors due to PTEN mutation. With the high content imaging system, we found that ~12% of the human kinome could directly or indirectly regulate Akt(ser473) phosphorylation. Of which, silencing of the ChoK, reduces Akt(ser473) phosphorylation significantly, suggesting its potential role as a regulator of PDK2.

## Results

### Silencing of Choline kinase A or B reduces Akt serine473 phosphorylation in MDA-MB 468 cells

**Figure 1:**
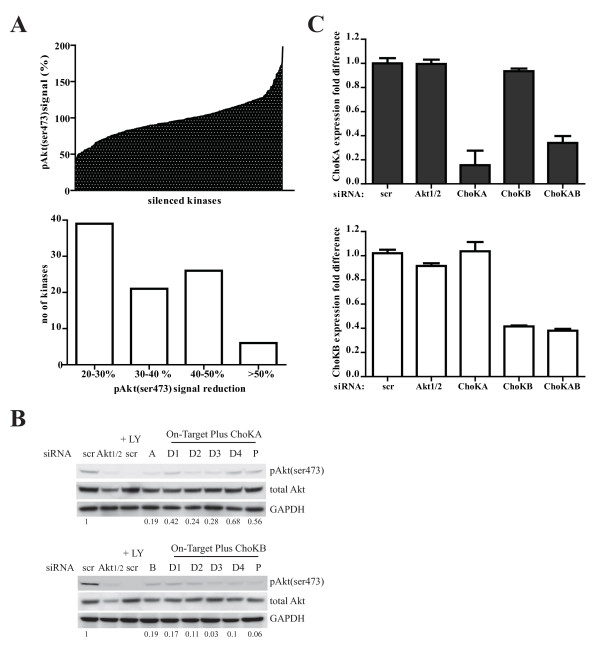
**Silencing of Choline kinase A or B reduced Akt(ser 473) phosphorylation in MDA-MB 468 cells**. A, The screen was set up using MDA-MB 468 cells as described in the method. The percentage pAkt(ser473) signal is calculated by obtaining total intensity (from fluorohore-Alexa 488) of the signal divided by total number of cells imaged. The reading is compared to the non-targeting siRNA transfected cells (scr, set to be 100%). B, Cells transfected with 50 nM pool (P) or deconvoluted (D1-4) ChoK A or B siRNA for 3 days and 30 μg whole cell lysate were subjected to western blot with the indicated antibodies. The signals obtained from the western blot analysis were quantified with Image J program and Akt(ser473) phosphorylation were normalized to total Akt. The ratios were compared to scr control (set as 1) indicated by the values below the blots. C, siRNA were transfected to cells as (B) for 2 days. 500 ng total RNA were reverse transcribed to cDNA subjected to real time PCR (ABI7500) with the appropriate primer sets. Transcript levels were normalized to the scrambled control (scr). The data is an average of triplicate experiments.

In search of kinases that could regulate Akt(ser473) phosphorylation, we utilized the human kinome siRNA library from Dharmacon on the MDA-MB 468 breast cancer cell line. After 779 serine, threonine, tyrosine and lipid kinases were systemically knocked down, cells were immunostained with anti-phospho-Akt(ser473) followed by anti-rabbit conjugated to Alexa 488 secondary antibody. Images were acquired using automatic high content screen fluorescent microscope (Discovery 1, Molecular Devices) and the level of cellular Akt(ser473) phosphorylation was analysed and quantified with MetaMorph software (data not shown). Our preliminary screen demonstrated that silencing of 12% of the human kinome resulted in a 20-60% reduction in Akt(ser473) phosphorylation (fig 1A) and these include mTor, PKCα and PI3K which are known to modulate Akt phosphorylation. Accordingly, silencing of 6 kinases resulted in more than 50% reduction in the phospho-serine (pAkt(ser473)) signal. Silencing of the lipid kinase, ChoK A resulted in 53% reduction of pAkt(ser473) signal which is one of the strongest inhibition in this screen. Silencing of the family member, ChoK B, also resulted in 46% reduction in the pAkt(ser473) signal.

The effects of ChoK A or B on Akt phosphorylation were validated using deconvoluted siRNAs as well as the more specific On-Target plus siRNA. As shown in fig 1B, silencing of both ChoK A and B resulted in strong reduction on pAkt(ser473) by the western blot analysis. Using real time PCR, successful knock down of ChoK A and B were confirmed (fig 1C).

### ChoK regulates Akt activity

**Figure 2:**
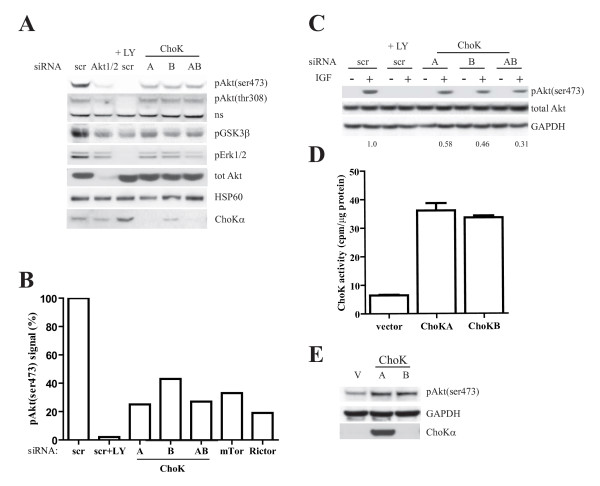
**Choline kinase regulates Akt activity**. A, MDA-MB 468 cells were transfected with 50 nM of the indicated pool siRNA. 3 days posttransfection, 50 μM LY294002 was added to scr transfected cells for 30 mins. Whole cell lysate were subjected to western blotting with the indicated antibodies. B, siRNA transfection and western blot were performed as (A). Percentage of pAkt(ser473) signal were calculated by quantifying the pAkt(ser473) normalized to total Akt on the immunoblot and compared to the scr control (set as 100%). C, MDA-MB 231 cells were transfected with 50 nM of indicated pool siRNA for 2 days. Cells were serum-starved overnight and stimulated with IGF for 15 mins. Whole cell lysates were harvested and western blot performed with the indicated antibodies. The values below the blot indicate the ratio of normalized pAkt(ser473) signals quantified using Image J to that of scr control (set as 1). D, MCF7 were transfected with 1 μg pcDNA vector, ChoK A or B plasmids for 24 h using Lipofectamine 2000 (Invitrogen). Cells were harvested and ChoK activity determined as described in the methods. E, MCF7 was transfected as in (D) and western blot with indicated antibodies.

Next, we addressed how the silencing of ChoKs might affect Akt signaling pathway. By immunoblotting for a number of proteins, we demonstrated that in ChoK-silenced cells, the level of pAkt(thr308) or total Akt protein remained unchanged (fig 2A). However strong reduction in GSK3β phosphorylation, an Akt downstream target, was observed. Inhibition of Akt phosphorylation by silencing of ChoKs resulted in reduced Erk phosphorylation, as seen with PI3K inhibitor, LY294002.

It has previously been demonstrated that the mTor complex 2 (mTORC2), of which Rictor is a component, is responsible for Akt(ser473) phosphorylation in a number of different cell systems [4]. To assess the contribution of the mTORC2 pathway in our system, mTor or Rictor were silenced (fig 2B). Immunoblotting with the pAkt(ser473) antibody demonstrated that ChoK A's effect on Akt(ser473) phosphorylation is equivalent to Rictor's, with more than 70% reduction following silencing of ChoK A or Rictor.

To demonstrate that the role of ChoK in Akt activation was not cell type specific, we performed the same silencing experiments on MDA-MB 231 cells. Two days after the siRNA transfection, the cells were serum-starved overnight and Akt activity was induced with the addition of Insulin-like Growth Factor (IGF) for 15 minutes. Here, in the cells with ChoK A or B or both silenced, stimulation with growth factor resulted in approximately 50% less Akt(ser473) phosphorylation compared to control cells (fig 2C).

To further demonstrate the regulation of Akt by ChoK, we overexpressed, either vector, ChoK A or B plasmids, in MCF7 cells. The overexpressed lipid kinases are active as shown in fig 2D. 24 hours posttransfection an increase in Akt(ser473) phosphorylation was observed (fig 2E).

### ChoK inhibitors inhibit ChoK activity and Akt phosphorylation

**Figure 3:**
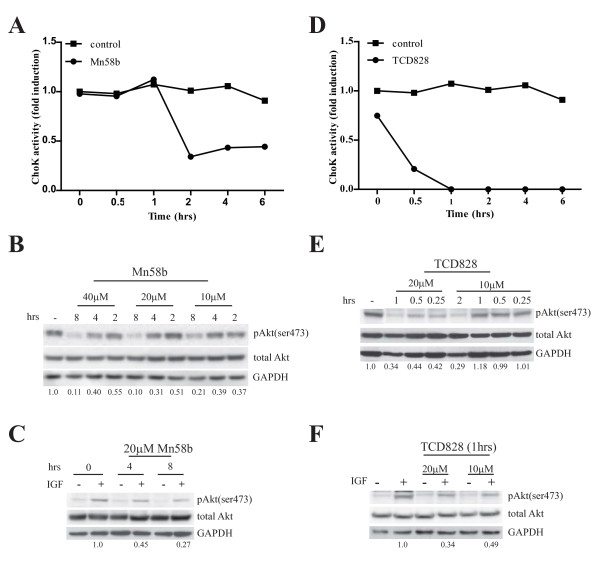
**ChoK inhibitors inhibit ChoK activity and Akt phosphorylation**. A, MDA-MB 468 cells were treated with 20 μM Mn58b for indicated time. Lysates were harvested and ChoK activity determined as described in methods. B, MDA-MB 468 and C, serum-starved MDA-MB 231 were treated with Mn58b for indicated time with the specified concentration. MDA-MD 231 cells were stimulated with IGF for 15 mins. 30 μg cell lysate were subjected to western blot with the indicated antibodies. D, MDA-MB 468 cells were treated with 20 μM TCD828. ChoK activity was determined as described in methods. E, MDA-MB 468 and F, serum starved MDA-MB 231 were treated with TCD828 for the indicated time with the specified concentration. MDA-MD 231 cells were stimulated with IGF for 15 mins. 30 μg cell lysate were subjected to western blot with the indicated antibodies. pAkt(ser473) signals were quantified using Image J program and normalized to respective total Akt signal. Values below the blots indicate the normalized Akt(ser473) phosphorylation compared to untreated control.

Next, we used small molecules inhibitors specific to ChoKα and lesser extent to ChoKβ to confirm ChoK activity is important for Akt phosphorylation. Two different inhibitors namely Mn58b and TCD828 were used to inhibit ChoK activity. Treatment with 20 μM of either inhibitors on MDA-MB 468 cells resulted in ~70% and ~85% reduction of ChoKα activity by 2 h for Mn58b and 0.5 h for TCD828 respectively (fig 3A, D). Western blotting showed a reduction of Akt(ser473) phosphorylation occurring in a dosage- and time-course dependent manner (fig 3B, E). Similar observations were made in MDA-MB 231 cells with IGF stimulation (fig 3C, F).

### ChoKα regulates Akt phosphorylation down stream of PI3K

**Figure 4:**
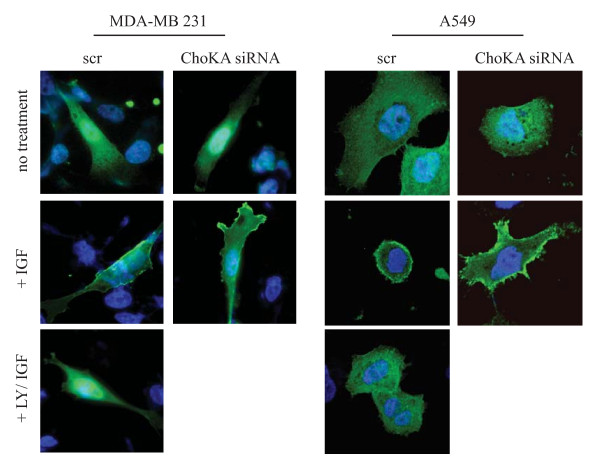
**ChoKα regulation on Akt activity is PI3K independent**. MDA-MB 231 and A549 cells were transfected with 50 nM of pool siRNA targeted to scr control or ChoK A. 2 days post transfection, cells were transfected with 0.5 μg PH-GFP construct for 24 h followed by 8 h serum starvation and 15 mins of IGF stimulation. 50 μM LY294002 was added to scr transfected cells for 30 mins prior to IGF stimulation. Cells were stained with Hoechst, fixed with paraformaldehyde and mounted for fluorescence microscopy.

In order to eliminate the possibility of ChoK having an indirect role on Akt phosphorylation for example through its action on PI3K, we tested the generation of PIP_3 _in ChoK-silenced cells. Here, we transfected a construct expressing the PH domain of Akt fused to GFP into two ChoK A - silenced cell lines, MDA-MB 231 and A549, a non-small cell lung carcinoma line. The cells were starved overnight followed by IGF stimulation. Using confocal microscopy, PH-GFP protein displayed a ring-like staining with plasma membrane localization in both cell lines after IGF stimulation. This is consistent with normal generation of PIP_3 _and the recruitment of PH-GFP following IGF stimulation (fig 4, left panel of each cell line). The ring-like localization of the PH-GFP was not observed when the cells were pre-treated with LY294002 (PI3K inhibitor). For ChoK A - silenced cells, the staining pattern were identical to control with plasma membrane localization after IGF stimulation (fig 4, right panel of each cell line). Taken together these data suggest that the role of ChoKα in mediating Akt phosphorylation is independent of PI3K.

### Mn58b treatment slowed tumor growth through the inhibition of Akt phosphorylation

**Figure 5:**
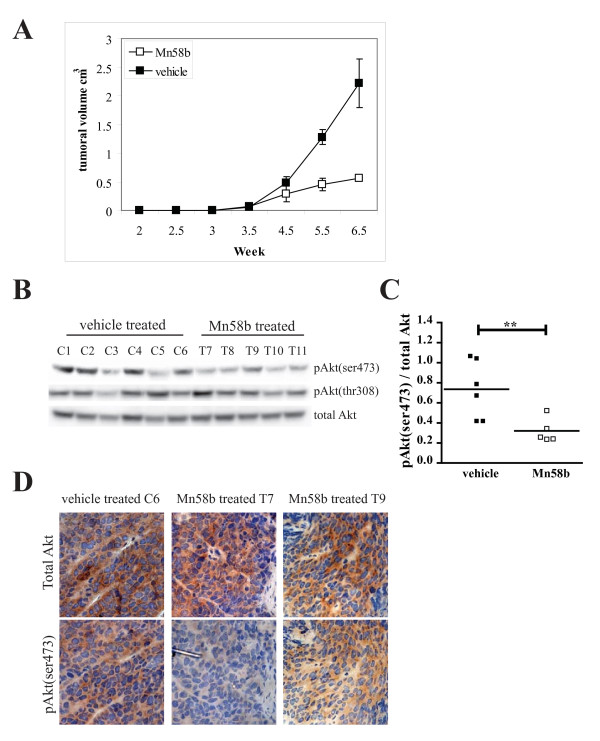
**Mn58b treatment slowed tumor growth through the inhibition of Akt(ser473) phosphorylation**. A, Immunosuppressed mice were injected with MDA-MB 231 cells on each flank and tumors were allowed to grow to 0.1 cm^3^. At week 3.5, Mn58b or vehicle, were administrated to the mice intraperitoneally and the growth of tumor monitored. B, 11 xenografts excised from the mice treated with vehicle (C1-6) or Mn58b (T7-11) were frozen and lysed for western blot with the indicated antibodies or D, fixed in paraformaldehyde and section for immunohistochemistry staining with anti-total Akt and anti-pAkt(ser473). C, pAkt(ser473) signal was quantified with Image J program and normalized to total Akt. The ratios were input as scatter plot and analyzed using unpaired t-test with Prism (GraphPad). ** p < 0.05.

To further consolidate the regulation of Akt(ser473) phosphorylation by ChoK *in vivo*, tumor xenografts treated with Mn58b were tested for the level of Akt phosphorylation. Immunosuppressed mice were injected with MDA-MB 231 cells on each flank and tumors were allowed to grow to 0.1 cm^3^. Mn58b or vehicle, were administered to eleven mice intraperitoneally and the growth of tumor monitored. As shown in fig 5A, tumor growth rate was significantly slowed upon treatment with Mn58b compared to vehicle control treated mice. Excised tumors from both vehicle and Mn58b treatment were fixed with formaldehyde or frozen immediately for immunohistochemistry staining and western blotting respectively. From the western blot (fig 5B), 4 out of 5 Mn58b treated tumors showed a reduction in the level of Akt(ser473) phosphorylation but not Akt(thr308), compared to vehicle treated tumors. Statistical analysis of the normalized phosphoAkt(ser473) signals from the western blot analysis revealed significant difference between the vehicle and Mn58b treated tumors with p values of 0.0075 (fig 5C). The decreased in Akt(ser473) phosphorylation correlated with small tumor size (fig 5A). This reduced Akt(ser473) phosphorylation after ChoK inhibitor treatment was confirmed using IHC staining with anti-total Akt and anti-phosphoAkt(ser473) (fig 5D). Mn58b treated tumor sections (T7 & T9) displayed similar total Akt level with low phosphorylation at the ser 473 site compared to the vehicle treated tumor sections (C6). These data demonstrate that inhibition of ChoKα *in vivo *results in attenuation of Akt(ser473) phosphorylation, substantiating a role for this lipid kinase in the regulation of Akt phosphorylation and tumor growth.

## Discussion

In this work, we used human kinome siRNA library to screen for kinases that positively regulate Akt phosphorylation at the ser473 residue in the breast cancer cell line, MDA-MB 468. MDA-MB 468 cells have an intrinsic PTEN mutation resulting in high endogenous Akt activity in the absence of growth factors. The systematic silencing of individual kinases in these cells with the RNA interference library allows us to identify kinases that alter Akt(ser473) phosphorylation. In combination with the high content screening microscope, we found a total of 92 kinases that upon knock down, resulted in 20 to 60% decrease in Akt(ser473) phosphorylation. In the screen setup, due to the "edge" effect of the 96 well plates, we noted that the standard deviation of these wells were high. Hence, these samples were not considered further. Regardless, the screen enables us look at 500 kinases and their effect on Akt phosphorylation.

Further validation had shown that ChoKα, plays an important role in regulating Akt(ser473) phosphorylation. Our data showed that ChoK is unlikely to act on the components upstream of Akt such as the PI3K signaling axis. This is showed by the ability of PH-GFP fusion protein to be recruited to the peripheral membrane in the presence of IGF stimulation in ChoK-silenced cells. These results demonstrated that PI3K is functional and able to generate PIP_3 _for the recruitment of both Akt and PDK1 as shown with the intact Akt(thr308) phosphorylation in these cells. Interestingly, other than the reported effects on Akt(ser473) phosphorylation, we also observed a decrease in Erk phosphorylation in ChoK-silenced cells. Since silencing of ChoK does not affect PI3K activity, it is unlikely that the reduced Erk phosphorylation is due to an inactivation of the upstream Ras. It is however possible that the reduction of Erk phosphorylation is due to yet unknown effects of this lipid kinase upon the Raf/MEK pathway, which will requires further investigation. Alternatively, the downstream effect on Erk signaling could arise from the cross talk between PI3K/Akt pathway and the Raf/MEK pathway, as shown with PI3K inhibitor, LY294002 treatment.

Although our data from both the RNAi silencing and small molecule inhibitor studies clearly demonstrate an interesting role of ChoK on Akt(ser473) phosphorylation, it is unlikely that the lipid kinase phosphorylate Akt(ser473) directly since our data with the ChoK inhibitors demonstrated a distinct lag time between ChoK activity inhibition and Akt phosphorylation. Only ~50% reduction in Akt(ser473) phosphorylation was observed when 70% of ChoKα activity was inhibited after 2 h of Mn58b treatment. A similar observation was made for TCD828 treated cells with a 56% reduction in Akt(ser473) phosphorylation after 0.5 h incubation with TCD828 which inhibited ~85% of ChoKα activity. In addition, we did not observe a physical interaction between Akt and ChoKα *via *co-immunoprecipitation (data not shown). Nonetheless, the evidence presented by xenograft regression with reduced Akt(ser473) phosphorylation and strong inhibition in Akt(ser473) phosphorylation after prolong treatment with ChoK inhibitors strongly support our data, suggesting a probable role of ChoKα as a regulator of PDK2, controlling the phosphorylation of Akt at ser473. Alternatively, the effect of ChoKα on Akt(ser473) phosphorylation could also arise through the inactivation of the Akt phosphatase. Previously, PH domain leucine-rich repeat protein phosphatase, PHLPP was identified by Gao *et al *as the phosphatase that dephosphorylate Akt1(ser 473) [22]. Further experiments will be required to definitively demonstrate these unexpected properties of ChoKα.

These findings are particularly exciting for two reasons. Firstly, there are several potential kinases that phosphorylate Akt(ser473). Of which, mTORC2 effect on Akt is significantly reproducible in many different cell types. In our work, we had shown that silencing of the lipid kinase, ChoK, resulted in reduced Akt(ser473) phosphorylation to a similar degree as observed following the silencing of Rictor, a member of the mTORC2 complex. Secondly, reminiscent of the regulators of the Akt pathway, there is evidence that ChoK can serve as marker for tumor progression. It has been shown that ChoK activity and its product, PCho, are increased in tumor cells relative to the normal cells [12,23-27]. This has been established in tumors of different tissue origins and in particular those derived from the breast [28,29]. It has also been demonstrated *in vivo *by NMR, where increase levels of PCho are frequently associated with cell malignancy [30-32]. All these results have established PCho as a malignancy marker with potential use in cancer diagnosis [28,33]. Our data demonstrate the presence of a novel cross talk between the lipid kinase and Akt pathway

Although the precise role of ChoK in these cancer cells is still not fully understood, it has been postulated that this lipid kinase is likely to be upregulated in order to provide lipid components for the actively dividing cancer cells. In addition, the PCho appears to induce mitogenic signaling, promoting cellular proliferation. Currently, there is an active effort in the development of ChoK inhibitors. Results from Mn58b, a well characterized ChoK inhibitor with *in vitro *and *in vivo *antiproliferative and antitumoral effect in mice xenografts provides strong support to this concept.

## Conclusions

Based on the information provided here and previous publications, we propose that ChoK displays oncogenic activity through activation of specific signaling pathways that impinge on cell proliferation and survival. One important signaling pathway affected is its interaction with Akt in cancer cells. However, we are uncertain of how this interaction regulates Akt other than it is required for ser473 phosphorylation. One probable hypothesis is that ChoK acts as an adaptor for a yet unidentified Akt(ser473) kinase. Alternatively, it would be interesting to determine if there is presence of any relationship between ChoK and mTORC2 activity.

## Methods

### Cell line and reagents

All cell lines were purchased originally from ATCC. MDA-MB 468, MDA-MB 231 and MCF7 were cultured in Dulbecco's modified Eagle's medium (DMEM) supplemented with 10% fetal calf serum. Cells were incubated in 37°C incubator with 10% carbon dioxide. ChoK A and B plasmids, monoclonal anti-ChoKα, Mn58b and TCD828 are kind gifts from Prof Lacal. All reagents unless specified are from Sigma-Aldrich (Saint Louis, MO).

### Human kinome siRNA screen setup

The 779 human kinase siRNA kinome library was supplied by Dharmacon as SMARTpool on 10 × 96 well plates. Non-Targeting siRNA or scrambled siRNA (scr) is used as negative control. 7500 MDA-MB-468 cells were seeded in 96 well plates the day before transfection. 70 nM siRNA were transfected using Oligofactamine according to manufracturer's instruction in triplicates. 72 hours post transfection, cells were fixed and proceeded to indirect immunofluorescent staining.

### Indirect Immunofluorescent labeling

After desired period of treatment, cells were washed once in PBS and fixed in 4% paraformaldehyde. Cells were permeabilised with 0.1% Triton X-100 followed by blocking with 3% BSA/PBS for 1 h and incubation with 1:250 anti-phospho-Akt(ser473) in 0.1% BSA/PBS overnight at 4°C. Subsequently, cells were washed followed by the addition of anti-rabbit secondary antibodies conjugated with Alexa Fluor 488 for 1 h. Nuclei were counterstained with 250 ng/ml H-33342 (Molecular Probes). Fluorescent images were collected and analysed using either Discovery-1 (Molecular Devices) or confocal microscope.

### Phospho-Akt(ser473) signal quantitation

Images of siRNA transfected cells after immunostaining with anti-phospho-Akt(ser473) were acquired using Discovery-1, high content screening fluorescent microscope, with 10× objectives. Three fields were imaged per well and total of 9 images were captured per kinase knock down. Images were analysed by MetaMorph. The phospho-Akt signal per cell per kinase knock down was calculated by obtaining total intensity (from the Fluorohore) of the signal divided by total number of cells imaged. This reading was compared to the non-targeting siRNA transfected cells (scr, set to be 100%) and background fluorescence reading (set to be 0%). Standard deviation was obtained from triplicate experiments.

### siRNA Transfection

siRNA targeting ChoKA, ChoKB, Akt1/2 and non-targeting control (scr) were purchased from Dharmacon (Dharmacon RNAi Technologies, Lafayette, CO) as SMARTpool or deconvoluted set of 4. Transfections using Oligofectamine (Invitrogen, San Diego, CA) were performed following the manufacturer's instructions.

### Semi-Quantitative PCR

RNA were isolated from cells using RNeasy kit from Qiagen. 500 ng total RNA was used to synthese complementary DNA using Superscript III first strand synthesis for RT-PCR (Invitrogen). 2 μl of the cDNA were loaded for Real time PCR using ABI 7500 with SYBR green mix (Applied Biosystems) and respective primer sets. ChoKA Forward: TAGGTTTGCCCTTGCATCTCA reverse: TCACACCCCAAGCTTCCTCTT ChoKB Forward: GTACATCCCAAGTCGGCCATT reverse: GCTCCTTGGTGAAAGGCATCT GAPDH Forward: CCCTTCATTGACCTCAACTACAT reverse: TCCTGGAAGATGGTGATGG

### Transfection

Transient transfections using Lipofectamine™ 2000 (Invitrogen) were performed following the manufacturer's instructions.

### Inhibitor treatment

Cells were seeded the day before on 6 well dish. Mn58b or TCD828 were added to the medium for indicated time and concentration. For MDA-MB 231, after incubation with inhibitors, 50 ng/ml of IGF was added for 15 mins before whole cell lysate were harvested for western blot.

### Western Blot

Cells were lysed in 1% triton lysis buffer. Protein concentration was determined using BCA assay (Thermo scientific). 30 μg of protein lysate were separated on 4-12% Tri-glycine gel (Invitrogen). Protein were transferred to PVDF membrane and immunoblotted with anti-phospho-Akt(ser473), antiphospho-Akt(thr308), anti-phospho-Erk1/2, anti-phospho-GSK3β (Cell Signaling Technology, Beverly, MA), anti-ChoKα, anti-HSP60, anti-tubulin, anti-GAPDH. Image J program was used to quantified the western blot signal. Phospho-Akt(ser473) signal were normalized to the respective total Akt. Difference in phosphorylation was obtained by comparing to scr control or untreated cells (set as 1).

### Choline kinase activity assay

ChoK activity were assayed as described in [34].

### In vivo Anti-tumor Assays

Human breast tumor xenografts were established by subcutaneous injection of tumor cells in each flank of nu/nu immunosuppressed mice. Mice were kept under standard laboratory conditions according to the guidelines of the Spanish government. Cells were resuspended in Dulbecco's modified Eagle's medium just before inoculation (2 × 10^6 ^cells/0.1 ml). When tumors reached a mean volume of 0.1 cm^3^, mice were randomized to control and treatment groups (6 mice per group). Treatments with Mn58b (and vehicle for control mice) were performed intraperitoneally for two week of treatmentseparated by 9 days. In the treatment weeks, the schedule was three doses of 2 mg/kg each, three times in the week. Tumors were monitored at least twice a week by measuring the major (*D*) and minor (*d*) diameters, and tumor volume was calculated as *V *= (*D *× *d*^2^)/2. The drug was well tolerated by the mice, with no significant effects on general appearance or behavior. Toxicity effects were confirmed by using hairy mice, treated under similar conditions of dose and schedule. No effects on fur, general appearance, or behavior were observed. No reduction in body weight was observed

### Immunohistochemistry staining

Tumors removed from mice were fixed and IHC performed according to [35].

## Abbreviations

ChoKA: Choline kinase A gene; ChoKα: Choline kinase A protein; PCho: phosphoryl Choline; ser473: serine 473; PDK1: phosphoinositide dependent protein kinase 1; NMR: nuclear magnetic resonance.

## Competing interests

The authors declare that they have no competing interests.

## Authors' contributions

BTC initiated the human kinome screen, identified and validated the role of Choline Kinase on Akt regulation. DGO constructed the overexpression Choline kinase plasmids, performed the Choline kinase activity assay and set up the xenograft model. ARM and JCL provided the inhibitors and Choline kinase antibodies as well as advices for both experiments and manuscript. The screen was initiated and performed under the supervision and funding of JD, CRUK and the work is continued under the support of AU, Institute of Medical Biology. The manuscript is drafted by BTC. All authors had read and approved the final manuscript
